# Impedance Cardiography in the Diagnosis of Congestive Heart Failure: A Systematic Review and Meta-Analysis

**DOI:** 10.7759/cureus.77461

**Published:** 2025-01-15

**Authors:** Christoph Müller

**Affiliations:** 1 Research, Marburg University, Marburg, DEU

**Keywords:** heart failure, impedance cardiography, meta-analysis, systematic review, wearable technology

## Abstract

According to current guidelines by the American Heart Association (AHA), the European Respiratory Council (ERC), and the European Society of Intensive Care Medicine (ESICM), the diagnosis of congestive heart failure is based on physical examination, laboratory assessment, and technological tests including echocardiography and chest X-ray. However, depending on different clinical variables, the assessment of BNP/NT-proBNP is generally recommended as the most sensitive method in making the diagnosis of congestive heart failure. Despite its high clinical utility, the measurement of BNP/NT-proBNP provides no information on the underlying pathophysiology or the hemodynamic state of the patient presenting. Impedance cardiography (ICG) enables clinicians to perform non-invasive continuous hemodynamic measurements to gain a more comprehensive view of the dynamics of heart failure. This systematic review and meta-analysis intend to investigate the relationship between different hemodynamic parameters measured with a bioimpedance device and levels of BNP/NT-proBNP to assess the usefulness of ICG in patients with acute heart failure. The present work was conducted according to the Preferred Reporting Items for Systematic Reviews and Meta-Analyses (PRISMA-P2015) guidelines. Electronic databases including PubMed, EMBASE, and GoogleScholar were searched for prospective studies investigating the relationship between BNP/NT-proBNP measurements and hemodynamic parameters in patients with heart failure. Statistical analysis including calculation of effect sizes, assessment of heterogeneity, and publication bias was performed using the software jamovi (jamovi project, 2024). From the initially identified 270 records, a total count of 11 articles met the eligibility criteria of the systematic review, of which nine studies were included in the meta-analysis. Summarizing the correlations between ICG parameters and BNP/NT-proBNP levels, a statistically significant relationship between the thoracic fluid content (TFC) (CC: 0.332, 95% CI: 0.184; 0.479, p 0.001), the cardiac index (CI) (CC: -0.312, 95% CI: -0.469; -0.155, p<0.001), stroke volume index (SVI) (CC: -0.369, 95% CI: -0.655; -0.083), and systolic time ratio (STR) (CC: 0.230, 95% CI: 0.117; 0.342, p<0.001) were observed. By summarizing the existing data on the relationship between hemodynamic parameters measured with ICG and levels of BNP/NT-proBNP, we could find substantial evidence for the utility of ICG in the diagnosis of heart failure. It seems to be particularly useful in differentiating shock states and guiding hemodynamic stabilization treatment with inotropes and vasopressors.

## Introduction and background

The diagnosis of acute and chronic heart failure can be made with great sensitivity based on the clinical examination and the assessment of BNP/NT-proBNP levels [1]. However, a more comprehensive understanding of the underlying pathophysiology requires further assessment with technical tests like echocardiography and chest x-rays. The hemodynamic state in patients with heart failure is investigated only in rare cases when patients are admitted to the emergency department, and transpulmonary or pulmonary-arterial thermodilution is accessible. In addition, the assessment of stroke volume (SV) and cardiac output (CO) can be conducted by applying the continuity equation during echocardiography [2]. While the clinical usefulness of thermodilution is restricted by its invasiveness, echocardiography can practically not be performed continuously and is associated with inter-observer variability [3]. Impedance cardiography (ICG) is a non-invasive technique, which allows for continuous hemodynamic measurements of different parameters like SV, CO, systolic time intervals, or thoracic fluid content (TFC) (Table 1).

**Table 1:** Cardiovascular parameters measured with ICG CI, cardiac index; CO, cardiac output; CVP, central venous pressure; MAP, mean arterial pressure; PCWP, pulmonary capillary wedge pressure; PEP, pre-ejection period; PCWP, pulmonary capillary wedge pressure; LVET, left-ventricular ejection time; STR, systolic time ratio; SV, stroke volume; ICG, impedance cardiography; TFC, thoracic fluid content; SV, stroke volume; SVI, stroke volume index

Parameter	Unit	Calculation
Blood flow		
SV	mL	
SVI	mL*m^-2^	SV/body surface area
CO	L*min^-1^	SV*heart rate
CI	L*min^-1^*m^-2^	CO/body surface area
Left cardiac work index	kg*m^-1^*m-^2^	(MAP-PCWP)*CI*0.0144
Contractility		
Velocity index	/1000 s^-1^	1000*(dz/dt)_max_/Z_0_
Acceleration index	/100 s^-2^	K*Ω*s^-2^
Systolic time intervals		
PEP	ms	Interval between electrocardiographic Q-wave until B-wave of (dz/dt)
LVET	ms	Interval between B-wave und X-wave of (dz/dt)
STR	ohne Einheit	PEP/LVET
Volume status		
TFC	K*Ω^-1^	1000*1/Z_0_
TFC index	/k*Ω*m^-2^	1000*1/Z_0_/body surface area
Vascular parameters		
Systemic vascular resistance	dyn*s*cm^-5^	((MAP - CVP)/CO)*80
Systemic vascular resistance index	dyn*s *cm^-5^*m^-2^	((MAP - CVP)/CI)*80

**Figure 1:**
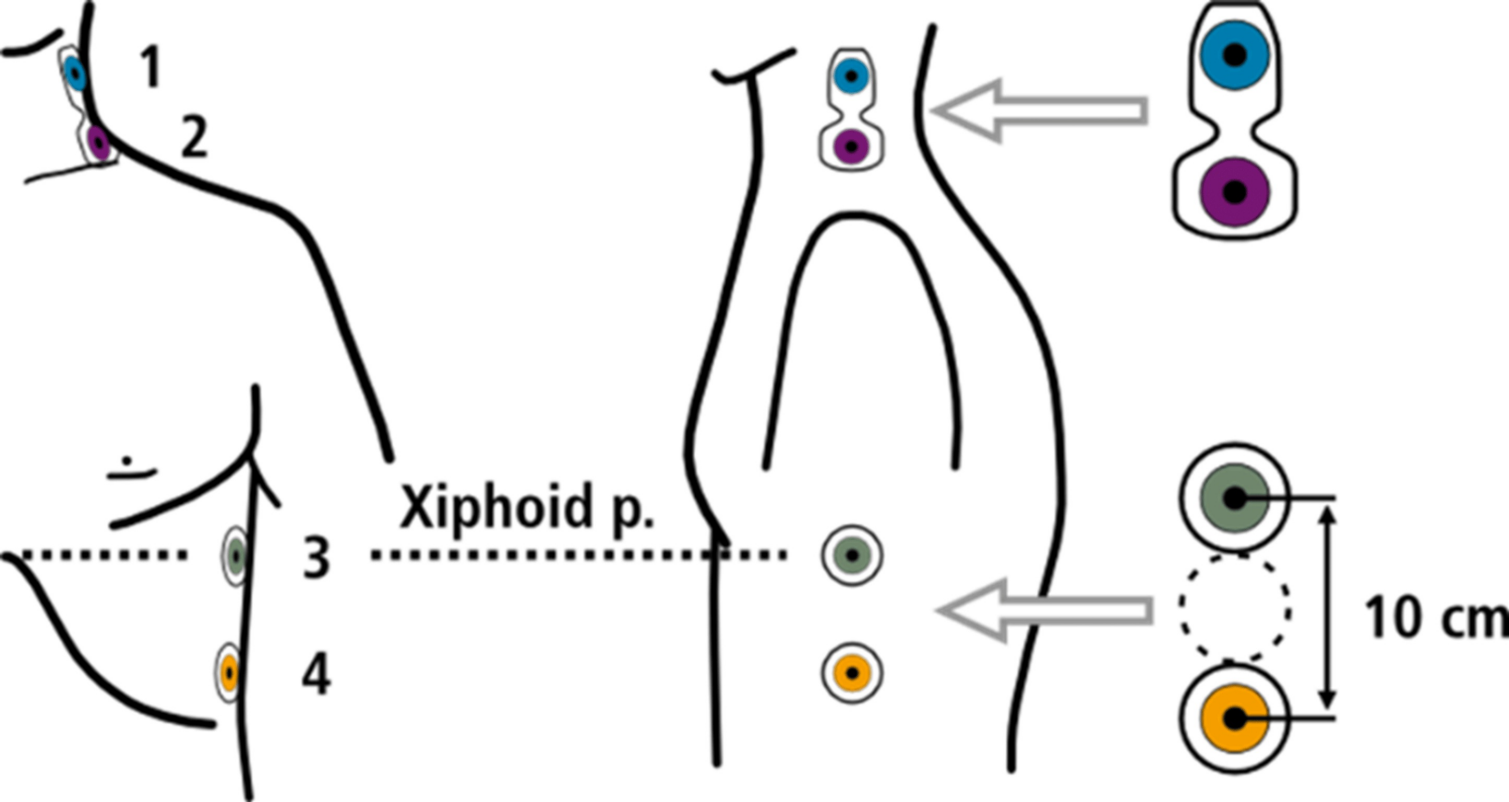
Electrode placement of the impedance cardiograph device (CardioScreen1000®, Ilmenau, Germany) Permission to distribute the image was received by medis Messtechnik GmbH.

**Figure 2:**
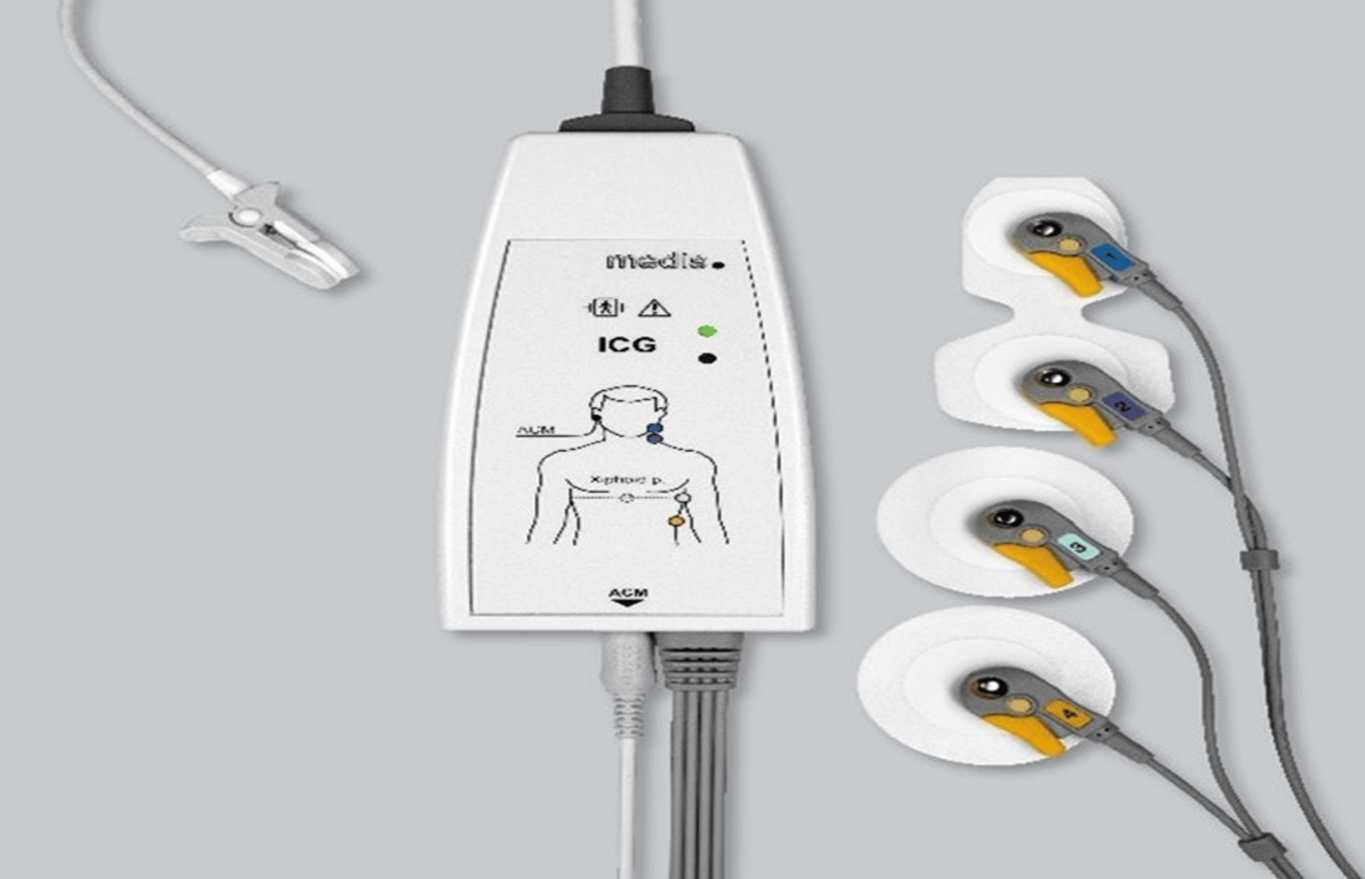
Impedance cardiograph device with electrodes and ACM ear clip (CardioScreen1000®, Ilmenau, Germany) ACM, arterial compliance modulation Permission to distribute the image was received by medis Medizinische Messtechnik GmbH.

**Figure 3:**
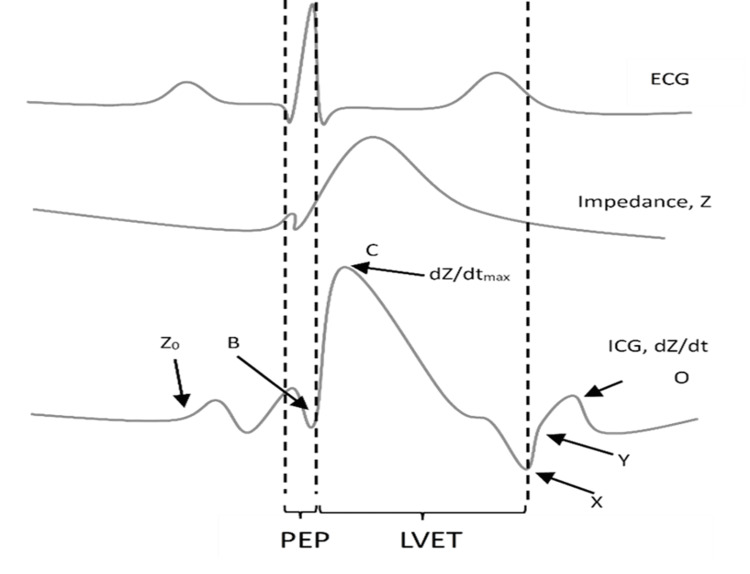
The time course of the ECG and impedance cardiogram, along with their characteristic turning points and the derived systolic time intervals B, opening of the aortic valve; C (dZ/dtmax), maximal systolic blood flow velocity; dZ/dt, maximal change in impedance; ECG, electrocardiogram; ICG, impedance cardiography; LVET, left-ventricular ejection time; O, closure of the mitral valve; PEP, pre-ejection period; X, closure of the aortic valve; Y, closure of the pulmonic valve; Z0, baseline impedance

Two dual electrodes are placed at the lateral side of the patient's neck and at the same side of the chest wall, which is illustrated for the ICG CardioScreen1000® (Medis, Ilmenau, Germany) in Figures 1, 2. To enable a more precise measurement of cardiac cycle intervals, an additional pulsoximetry sensor is attached to one ear lobe for recording pulse volume curves by infrared light. The technique helps in detecting the true X-point (Figure 3) of the ICG to determine the end of the LVET.

The outer electrodes produce a low-amplitude alternating current, which is sensed by the inner electrodes, and is used to measure the change in thoracic impedance over time. Its first derivative is used to extract the maximal change of thoracic impedance (dz/dt)_max_, which is used to calculate SV, CO, stroke volume index (SVI), systolic time intervals, or TFC [4].

The first formula for the calculation of SV was proposed by Kubicek et al. (1970) [5] in which (dz/dt)_max_ is multiplied by the left ventricular ejection time (LVET), the distance between the electrodes, the characteristic impedance of blood and divided by the baseline impedance: SV=p*L_0_^2^*Z_0_^-2^*(dz/dt)_max_*ET, where ρ=impedance of blood (Ω·cm), L₀=distance between electrodes (cm), Z₀=thoracic baseline impedance (Ω), (dz/dt)_max_=maximum of the first derivatives of thoracic impedance, and ET=ejection time [s].

This formula was modified by Sramek and Bernstein (1986) [6] so that it would account for patients' height and body weight. Most impedance cardiographs apply this equation to calculate SV and its derived parameters: SV=δ*(0.17*H)^3^/4.25*Z_0_*(dz/dt)_max_*ET, where δ=correction variable for bodyweight; H_0_=height; Z_0_ (cm)=thoracic baseline impedance (Ω); (dz/dt)_max_=maximum of the first derivatives of thoracic impedance; LVET=ejection time [s].

In addition to hemodynamic parameters, most impedance cardiographs allow for the measurement of systolic time intervals, which are determined by combining electrocardiographic and impedance cardiographic signals (Figure 3). The onset of the pre-ejection period (PEP), which consists of the electromechanical delay and the isovolumetric contraction period, starts with the electrocardiographic Q-wave and ends with the B-wave observed in the ICG curve. The B-wave reflects the opening of the aortic valve and marks the onset of the LVET, which ends with the X deflection that is related to aortic valve closure [7].

Several studies have validated ICG as a hemodynamic monitoring technique by showing a strong correlation with the reference method of thermodilution [8-10] and demonstrating a high retest reliability [11]. The clinical applications of ICG consist of monitoring cardiovascular pathologies like arterial hypertension or heart failure, the evaluation of shock states, or the optimization of pacemaker treatment. Although ICG is not recommended by current guidelines, its application seems particularly useful in the diagnosis and management of congestive heart failure. The measurement of thoracic bioimpedance not only allows for the evaluation of hemodynamic parameters like SV and CO but also enables clinicians to estimate the degree of fluid retention by determining the reciprocal of thoracic baseline impedance [12], which is referred to as the TFC; TFC=1000*1/Z_0_, where TFC=thoracic fluid content (k*Ω^-1^); Z_0_=thoracic baseline impedance (Ω).

The assessment of ICG parameters, including TFC and SV, therefore, appears to be of high clinical utility in the context of congestive heart failure. It may help to determine the pathophysiology as well as the degree of congestion and may thereby support clinicians to guide treatment.

Evaluating its usefulness in the setting of acute heart failure, a statistically significant relationship between ICG parameters and BNP/NT-proBNP levels as the recommended diagnostic method can be expected. 

We, therefore, searched for studies investigating the relationship between ICG parameters and BNP/NT-proBNP levels at the time of hospital admission. Theoretically, a positive correlation between TFC and BNP/NT-proBNP and an inverse relationship between SV and its related parameters with BNP/NT-proBNP can be hypothesized in patients with heart failure.

## Review

Methods

Eligibility Criteria

This systematic review and meta-analysis were conducted using the Preferred Reporting Items for Systematic Reviews and Meta-Analyses (PRISMA-P 2015) [13] checklist. Only studies with either a prospective observational or interventional study design were considered. Studies were included if the study population consisted of at least 20 participants to ensure a significant amount of statistical power, a measurement with ICG was performed, and levels of BNP/NT-proBNP were assessed. Assessment of BNP/NT-proBNP was performed with laboratory methods, usually radioimmunoassays. Articles were excluded if they were reviews, letters or comments, editorials, case reports or case series, and did not meet the above-mentioned criteria. No restrictions were made considering language or publication bias.

Data Sources and Search Strategy

A comprehensive literature search using the electronic databases PubMed, EMBASE, and GoogleScholar until October 31 2024 was conducted. The search strategy included the combination of search terms with Boolean operators and search by proximity. The keywords and Medical Subject Heading (MeSH) terms “impedance cardiography” and “BNP” OR “NT-proBNP” were used. References were exported with the Citavi© software (Lumivero, Denver, USA) and duplicates were removed.

Study Selection and Data Extraction

The identified records were assessed for eligibility by reading the titles and abstracts of each article. If the articles were eligible, the full text was obtained and investigated for inclusion in the systematic review. Each article was then assessed for data to be included in the meta-analysis. Exported data, which were used for meta-analysis, included correlation coefficients and sample size of each study. Quality assessment of the included studies was performed with the Quality Assessment Tool for Observational Cohort and Cross-Sectional Studies (NIH, 2017) [14,15]. A score of less than 50% of the total was defined as a high risk of bias for which studies were excluded from the meta-analysis.

Statistical Analysis

Summary effect size: The meta-analysis was performed with the online software jamovi© (jamovi project, 2024) [16]. Statistical analysis focused on the correlation between BNP/NT-proBNP levels and hemodynamic parameters measured by ICG. The correlation coefficient of each study was converted to the Fisher's z scale, and the summary effect size was calculated for the transformed value. For each included study, the correlation coefficient and the sample size were extracted to calculate the summary effect size. All studies assessed the BNP/NT-proBNP levels and ICG parameters at the time of admission. A random-effects model was chosen to calculate the summary effect size because of the expected interstudy variability. Statistical significance was assumed for a p≤0.05.

Heterogeneity testing: Different measures of heterogeneity were calculated to evaluate the variance among the included studies, the need for subgroup analysis, and the general interpretability of the summary effect size. Cochran's Q reflects the weighted sum of the squared differences between the observed effects from the weighted average effect and quantifies the deviation of each individual study from the mean effect size. The I^2^ statistic measures the proportion of the observed variance explained by the real differences of the individual effect sizes. According to Higgins et al. (2003) [17], heterogeneity can be described as low (I^2^<25%), moderate (25%≤I^2^≤75%), and high (I^2^>75%). In addition, studentized residuals and Cook's distance are calculated to examine if any statistical outliers may have a substantial effect on the general effect size. Studies with a studentized residual larger than 100×(1-0.05/(2×k))th percentile of the normal distribution, and a Cook's distance larger than the median plus six times the interquartile range of Cook's distance, are considered statistical outliers.

Publication bias analysis: To assess the probability that selective publication may have influenced the results of the present study, publication bias analysis was performed. The funnel plot provides a graphical display of the effect size plotted against the standard error of each study and should be symmetrically distributed if no publication bias is involved. The Begg and Mazumdar rank correlation test [18] and Egger's regression test [19] are used to quantitatively test for the presence of publication bias.

Results

Study Selection

**Figure 4:**
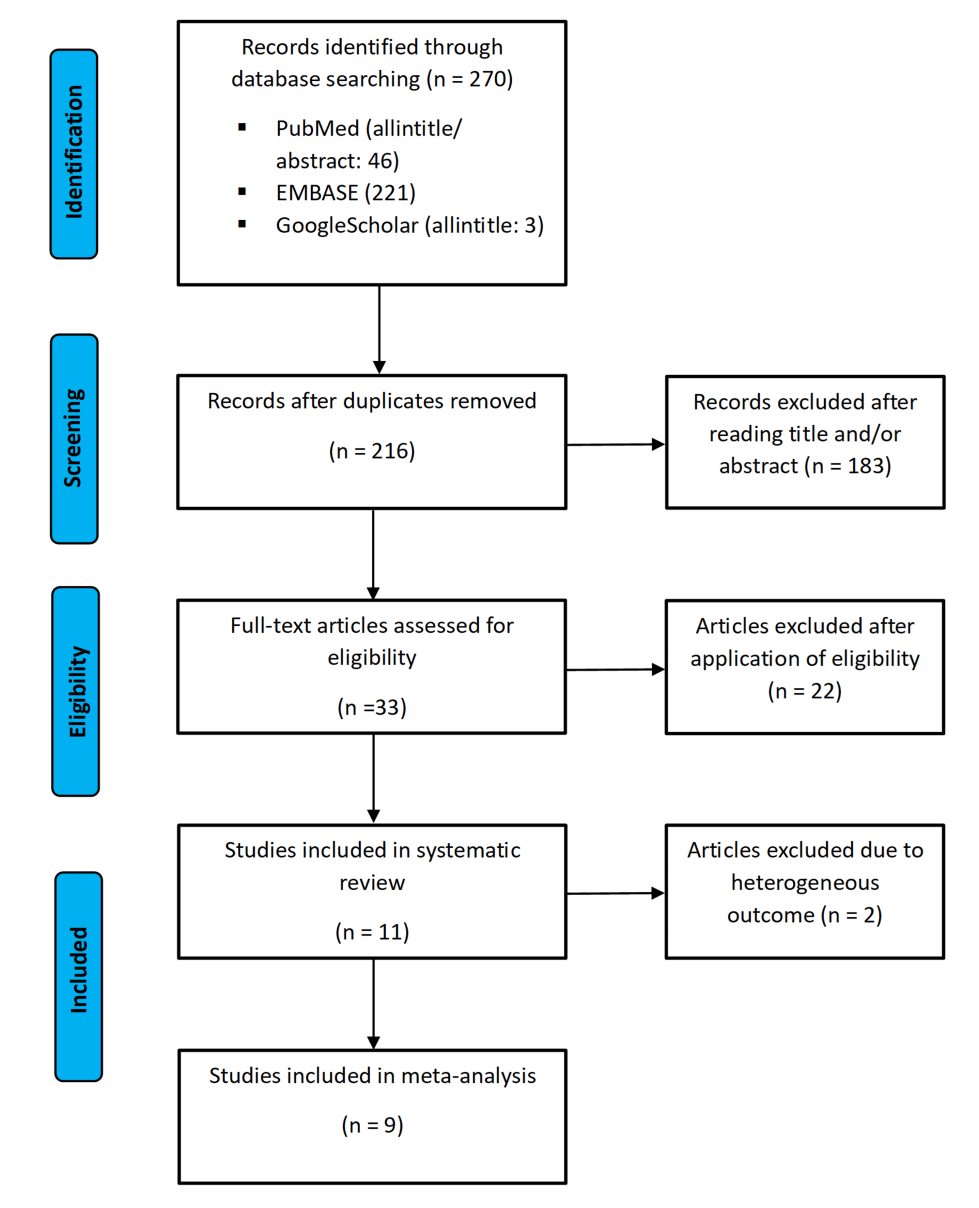
Flow chart of the study selection according to PRISMA PRISMA, Preferred Reporting Items for Systematic Reviews and Meta-Analyses

The process of study selection is illustrated in the flow chart in Figure 4. There was a total count of 270 records after the initial search including 46 from PubMed, 221 from EMBASE, and three from GoogleScholar. After the removal of duplicates, the remaining 216 records were then screened for eligibility. Finally, 11 studies met the criteria for the systematic review, of which nine studies were included in the meta-analysis.

Overview of Included Studies

The 11 included studies were conducted in six countries and were published between January 2008 and January 2024 (Table 2). Overall, the study population consisted of 1374 participants with a balanced male-to-female ratio and an average age of 67.42±4.53 years. The included studies applied either a prospective observational or prospective interventional study.

**Table 2:** Summary of included studies investigating the relationship between ICG parameters and BNP/NT-proBNP levels in patients with heart failure 6-MWT, 6-minute walk test; ACI, acceleration index; BNP, brain natriuretic peptide; BIVA, bioimpedance vector analysis; CHF, congestive heart failure; CI, cardiac index; cTnT, c Troponin T; FACIT-F, Functional Assessment of Chronic Illness Therapy - Fatigue; ICG, impedance cardiography; KDQOL, Kidney Disease Health-Related Quality of Life - Short Form 36; LCW, left cardiac work; LCWI, left cardiac work index; n, number of participants; NT-proBNP, N-terminal pro-brain natriuretic peptide; PEP, pre-ejection period; QIDS-SR16, 16-item Self-Reported Quick Inventory of Depressive Symptomatology; R, resistance; Ra, reactance; SI, stroke volume index; STR, systolic time ratio; SVR, systemic vascular resistance; SVRI, systemic vascular resistance index; LVEF, left ventricular ejection fraction; NYHA, New York Heart Association; TBW, total body water; TPRI, total peripheral resistance index; TTE, transthoracic echocardiography; TFC, total fluid content; TFCI, total fluid content index; VI, velocity index

Study ID			Participants			Intervention			Bioimpedance device	
Author (year)	Journal	Country	n (% female)	Age	Symptoms	Study design	Treatment intervention	Outcome	Name (company)	ICG parameters
Havelka et al. (2008) [20]	The Journal of Emergency Medicine	USA	54 (54%)	Median age: 74.90	Dyspnea due to congestive heart failure	Prospective observational study	ICG measurement and BNP levels at the time of admission to a tertiary care hospital	Relationship between BNP levels and CI, SVR, TFC at the time of hospital admission	Bio-Z ICG Monitor (Cardio Dynamics, San Diego, CA	CI, SVR, TFC
Parrinello et al. (2008) [21]	Journal of Cardiac Failure	Italy	172 (59%)	Mean age: 68.7 ± 5.1	Acute shortness of breath on exertion or at rest	Prospective interventional study	Treatment of acute heart failure, BNP levels at admission, segmental and whole-body BIA at admission and discharge	BNP level at admission, whole-body and segmental resistance/reactance at admission and discharge, relationship between BNP and BIA parameters	BIA-101 (Akern Srl, Florence, Italy)	Whole-body and segmental resistance/reactance
Balak et al. (2009) [22]	Kardiologia Polska	Poland	30 (10%)	Mean age: 53 ± 6	CHF due to ischemic or idiopathic-dilated cardiomyopathy in stable condition	Prospective observational study	ICG measurement, assessment of BNP levels at the time of hospitalization	Comparison of biometric data, clinical status, LVEF, 6-MWT, BNP, TFC, relationship between BNP and TFC	Task Force® Monitor 3040i (CNSystems Medizintechnik GmbH, Graz, Austria)	TFC
Malfatto et al. (2010) [23]	European Journal of Heart Failure	Italy	120 (26%)	Mean age: 71 ± 9	Symptoms of chronic systolic heart failure in stable condition	Prospective observational study	ICG measurement, assessment of BNP levels of patients presenting at an outpatient heart failure center	Comparison of biometric, clinical, echocardiographic, and ICG parameters, relationship between diastolic dysfunction, mitral regurgitation, BNP, and TFC	Bio-Z ICG Monitor (CardioDynamics, San Diego, CA, USA); Niccomo™ (Medis Medizi-nische Mess-technik GmbH, Jena, Germany)	TFC
Chen et al. (2014) [24]	International Journal of Clinical and Experimental Medicine	China	99 (76%)	Mean: 62.3 ± 11.2	Acute myocardial infarction planned for myocardial revascularization	Prospective observational study	ICG measurement, echocardiography and assessment of BNP, NT-proBNP and cTnT 2 days after surgery	Relationship between BNP, NT-proBNP, cTnT, and ICG and echocardiographic parameters	Bioz (Cardio-Dynamics, San Diego, CA, USA)	SV, SVI, CO, CI, SVR, SVRI, VI, ACI, PEP, LVET, STR, LCWI, TFC
Génot et al. (2015) [25]	American Journal of Emergency Medicine	France	37 (46%)	Mean: 71.5 ± 13.6	Acute dyspnea presenting to the emergency department	Prospective observational study	BIVA measurement, echocardiography, assessment of BNP levels	Comparison of biometric, clinical, echocardiographic, and BIVA parameters, relationship between BNP and BIVA parameters, prediction of acute heart failure by BIVA parameters	Z-Metrix (BioparhΩm, Bourget-du-Lac, France)	R, Ra, TBW, ECW
Sadauskas et al. (2016) [26]	Medical Science Monitor	Lithuania	60 (40%)	Mean: 67.9 ± 3.4	Symptoms of acute heart failure without sign of acute myocardial infarction	Prospective observational study	ICG measurement, assessment of BNP, echocardiography	Comparison between ICG parameters for different heart failure groups according to echocardiography and NYHA classification	Niccomo™ (Medis Medizinische Messtechnik GmbH, Jena, Germany)	CI, CO, LCW, LCWI, LVET, PEP, SI, STR, SV, TFC, TFCI
Sadauskas et al. (2018) [27]	Medical Science Monitor	Lithuania	120 (50%)	Mean age: 68.9 ± 2 .2	Clinical signs of acute heart failure	Prospective interventional study	Treatment of heart failure in the intensive care unit, ICG measurement and BNP levels at admission and discharge of patients, 6-months follow-up	Difference between ICG parameters after treatment of heart failure, relationship between BNP and ICG parameters at admission, survival curve after 6 months	Niccomo™ (Medis Medizinische Messtechnik GmbH, Jena, Germany)	CO, LCW, LCWI, SI, STR, TFC, TFCI
Niu et al. (2019) [28]	Medical Science Monitor	China	160 (44%)	Mean: 58.3 ± 15.4	Chronic heart failure due to coronary artery disease after off-pump coronary bypass graft	Prospective interventional study	ICG measurement, assessment of BNP, echocardiography	Comparison of ICG parameters between stages of heart failure and pre-/post-treatment, relationship between ICG parameters and BNP levels	BioZ Dx Impedance Cardiograph (Cardio Dynamics, San Diego, CA, USA)	ACI, CI, CO, LVET, PEP, SI, SV, SVR, TFC
Ališauskas et al. (2022) [29]	Medical Science Monitor	Lithuania	301 (45%)	Mean: 71.9 ± 6.7	Symptoms of chronic heart failure diagnosed by physical examination, NT-proBNP, chest radiography, and TTE	Prospective interventional study	ICG measurement, assessment of BNP, echocardiography before and after decongestive therapy	ICG parameters at the time of admission and discharge, correlation between ICG parameters, NT-proBNP, LVEF, and 6MWD, long-term clinical outcome	Niccomo™ (Medis Medizinische Messtechnik GmbH, Jena, Germany)	CI, CO, LCW, LCWI, LVET, PEP, SI, STR, SV, TFC, TFCI
Gregg et al. (2024) [30]	Journal of Investigative Medicine	USA	21	Median: 56.5 (non-CKD), 70.0 (CKD)	Normal or impaired renal function with or without hypervolemia, symptoms of chronic illness assessed with the FACIT-F, QIDS-SR16, KDQOL	Prospective observational study	ICG measurement, blood pressure, levels of BNP and NT-proBNP, FACIT-F, QIDS-SR16, KDQOL	Comparison of biometric, clinical, ICG parameters, BNP, and NT-proBNP between CKD and non-CKD, relationship between BNP or NT-proBNP with ICG parameters and blood pressure, relationship between reported burden of disease with blood pressure and ICG parameters	NICOM (Cheetah Medical, Inc. Wilmington, DE, USA)	CI, ECW, TPRI

Quality Assessment

**Figure 5:**
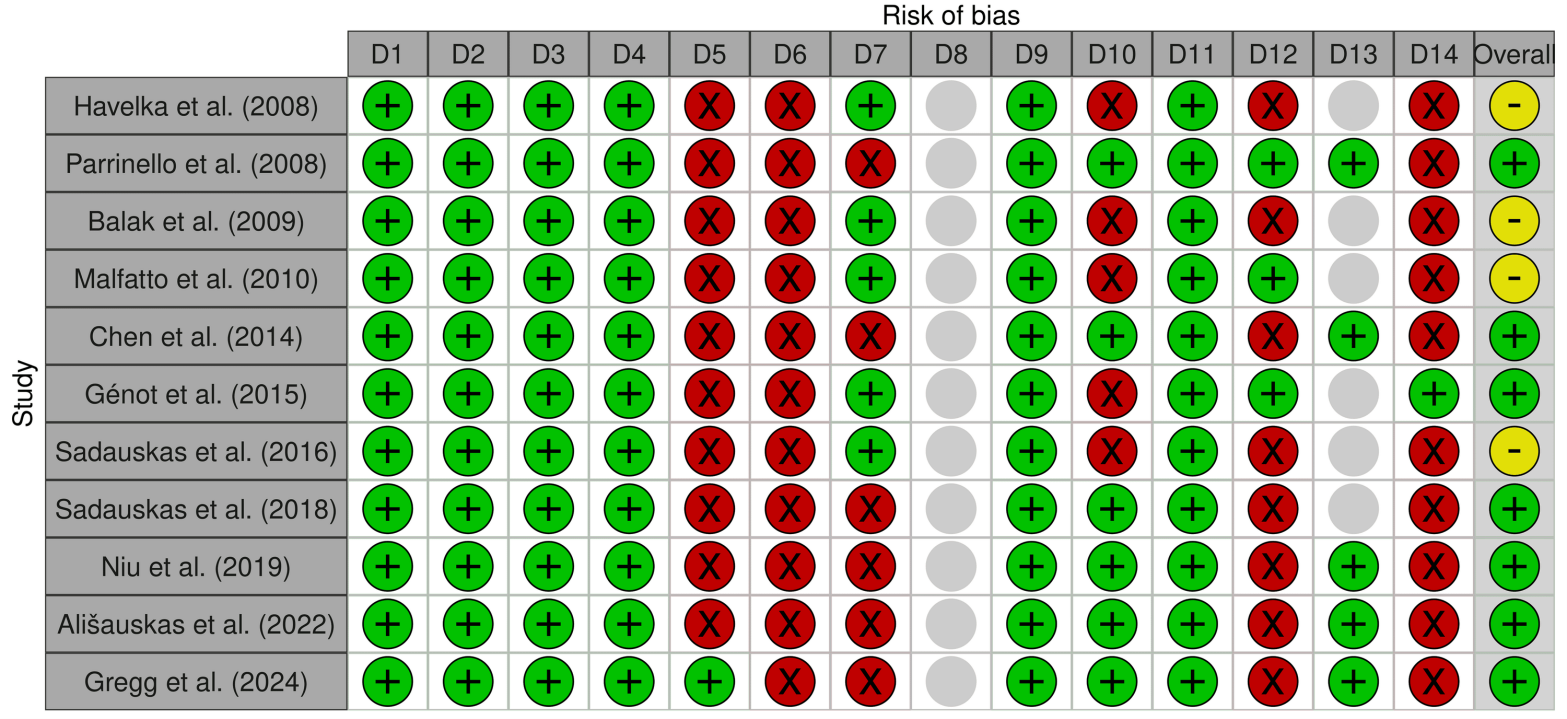
Illustration of the risk of bias assessment using the Quality Assessment Tool for Observational Cohort and Cross-Sectional Studies with the robvis (Risk-Of-Bias VISualization) tool D1, research questions clearly stated; D2, study population clearly specified and defined; D3, participation rate of eligible persons at least 50%; D4, all subjects from the same or similar population and inclusion/exclusion criteria specified; D5, sample size justification provided; D6, exposures of interest measured prior to outcome measurement; D7, timeframe sufficient to expect an association between exposure and outcome; D8, different levels  of exposure; D9, independent variables clearly defined, valid, reliable and implemented consistently; D10, exposure assessed more than once over time; D11, dependent variables clearly defined, valid, reliable and implemented consistently; D12, outcome assessors unaware of the exposure status of participants; D13, loss to follow-up 20% or less; D14, confounding variables measured and adjusted for impact on outcome [20-30] Red, high risk of bias; yellow, moderate risk of bias; green, low risk of bias; Grey, not applicable

All included studies were qualitatively assessed using the Quality Assessment Tool for Observational Cohort and Cross-Sectional Studies (NIH, 2014) [18], which includes 14 categorical questions (D1-D14). Each answer was scored for low or high risk of bias and an overall evaluation for each study was given. A graphical display of the results was provided using the robvis (Risk-Of-Bias VISualization) [19] tool in Figure 5. Although there was some concern with regard to most study designs being observationally conceptualized and lacking control groups, all identified studies seemed to have sufficient quality for the purpose of this systematic review and meta-analysis (Figure 5).

Meta-Analysis

Summary Effect Size

**Figure 6:**
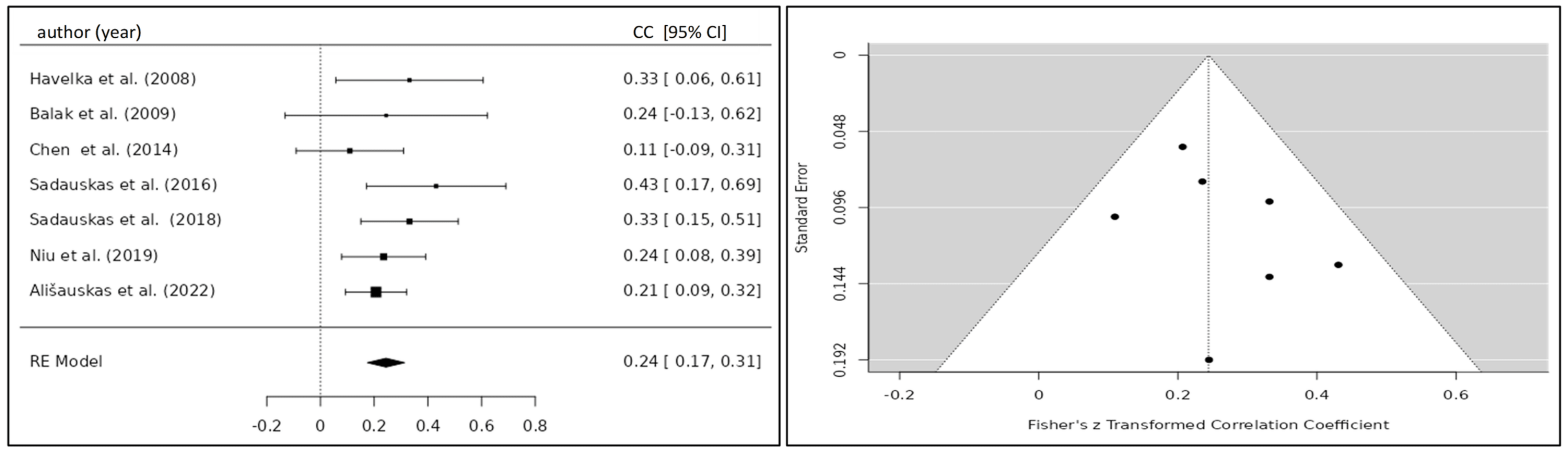
Forest plot of all included studies investigating the correlation between TFC and BNP/NT-proBNP. Funnel plot of all included studies investigating the correlation between TFC and BNP/NT-proBNP CC, correlation coefficient; CI, confidence interval; RE, random-effects model

**Figure 7:**
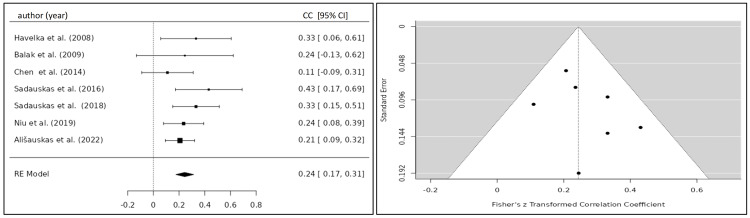
Forest plot of studies investigating the correlation between TFC and BNP/NT-proBNP excluding one study associated with significant heterogeneity. Funnel plot of studies investigating the correlation between TFC and BNP/NT-proBNP excluding one study associated with significant heterogeneity CC, correlation coefficient; CI, confidence interval; RE, random-effects model; TFC, thoracic fluid content

**Figure 8:**
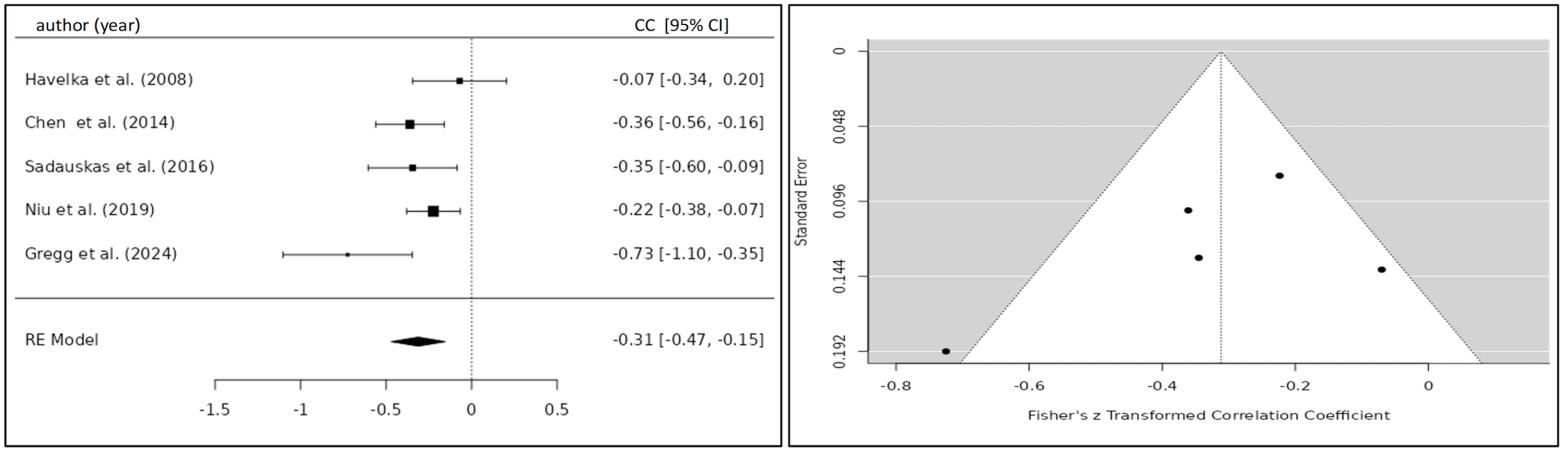
Forest plot of all included studies investigating the correlation between SVI and BNP/NT-proBNP. Funnel plot of all included studies investigating the correlation between SVI and BNP/NT-proBNP CC, correlation coefficient; CI, confidence interval; RE, random-effects model; SVI, stroke volume index

**Figure 9:**
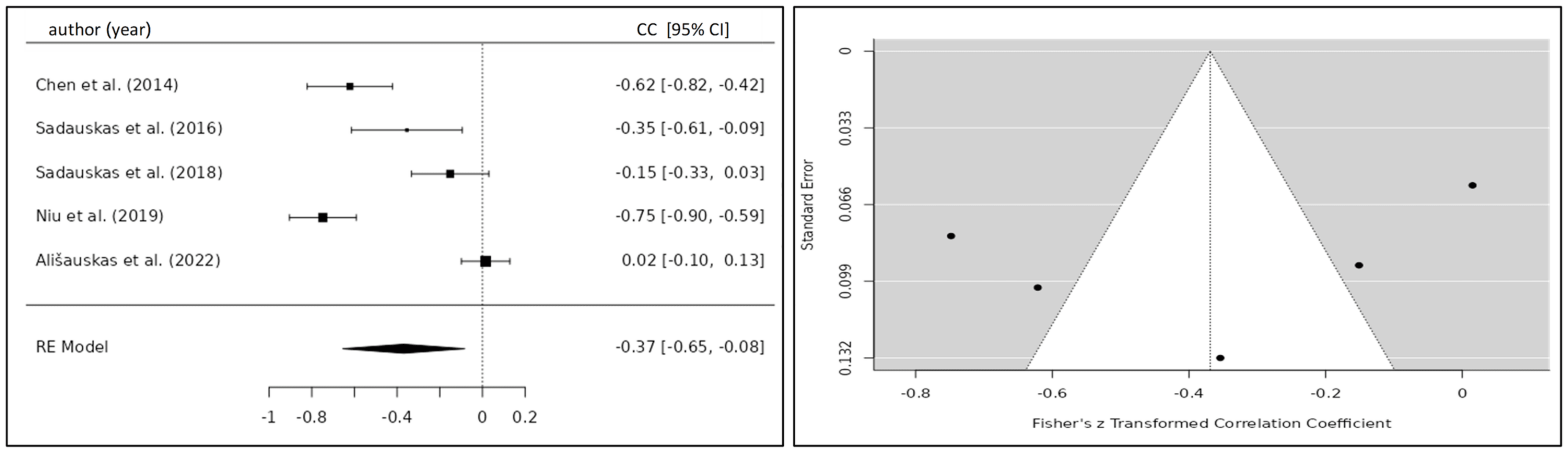
Forest plot of all included studies investigating the correlation between CI and BNP/NT-proBNP. Funnel plot of all included studies investigating the correlation between CI and BNP/NT-proBNP CC, correlation coefficient; CI, confidence interval; RE, random-effects model

**Figure 10:**
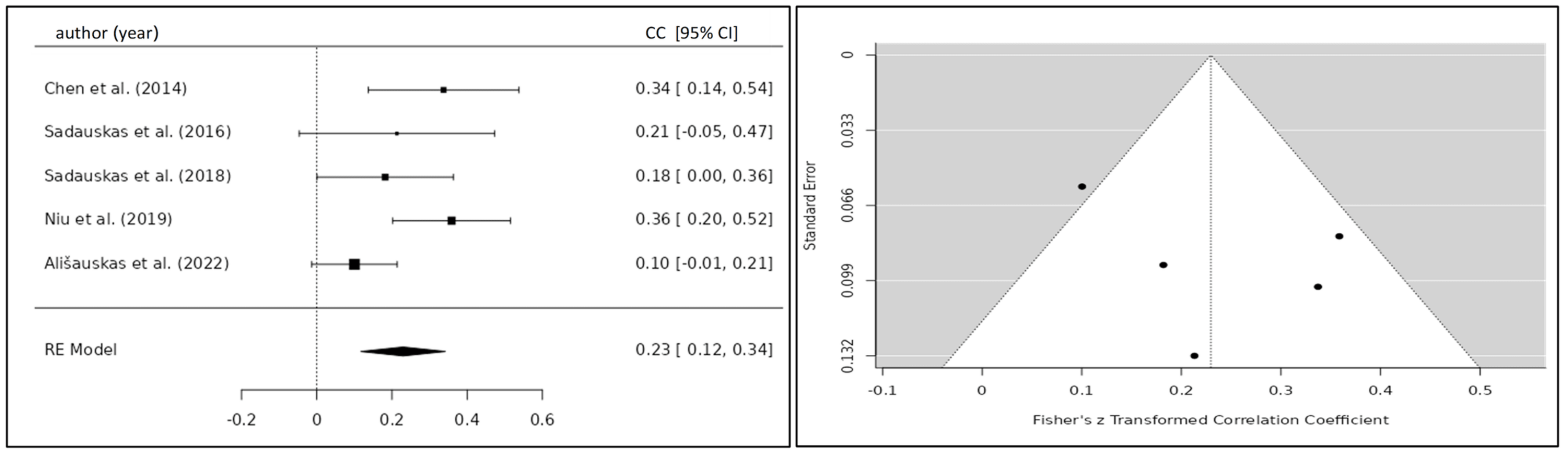
Forest plot of all included studies investigating the correlation between STR and BNP/NT-proBNP. Funnel plot of all included studies investigating the correlation between STR and BNP/NT-proBNP CC, correlation coefficient; CI, confidence interval; RE, random-effects model; STR, systolic time ratio

The correlation coefficient of the TFC and BNP/NT-proBNP for eight eligible studies was 0.332 (95% CI 0.184; 0.479) yielding a statistically significant result (p<0.001) (Figure 6) [20,22-24,26-29]. After the exclusion of one study, which caused significant heterogeneity, the correlation coefficient was 0.244 (95% CI 0.175; 0.313) (Figure 7) [20,22-27]. Five studies investigated the relationship between the SVI and BNP/NT-proBNP levels for which a summary correlation coefficient of -0.369 (95% CI -0.665; -0.083; p=0.011) was calculated (Figure 8) [20,24,26,28,30]. The correlation coefficient of BNP/NT-proBNP with cardiac index (CI) was -0.312 (95% CI -0.469 to -0.155), and with systolic time ratio (STR), it was 0.230 (95% CI 0.117 to 0.342), both yielding statistically significant results with a p-value <0.001 (Figures 9, 10) [24,26-29].

Heterogeneity Testing

The calculation of I^2^ and tau^2^ indicated a significant amount of heterogeneity among the included studies for the calculation of the summary correlation coefficient between TFC and BNP/NT-proBNP levels (Q(7)=32.4324, p<0.0001, tau²=0.0331, I²=78.0063%), which was reduced after exclusion of one study (Q(6)=5.440, p=0.489, tau²=0.0047, I²=0%). Statistically significant heterogeneity of the included studies was observed for the SVI (Q(4)=73.690, p<0.001, tau^2^=0.0975, I^2^=92.84%). A moderate, albeit not statistically significant, amount of heterogeneity was observed for CI (Q(4)=8.921, p<0.063, tau²=0.0167, I²=54.29%) and STR Q(4)=8.698, p<0.069, tau²=0.0084, I²=52.7%). The variance between studies might be explained by the application of different ICG devices and the varying sample size among the included studies.

Publication Bias Analysis

Neither the Begg and Mazumdar rank correlation nor the Egger's regression test showed any evidence of publication bias for TFC (p=0.533; p=0.927), SVI (p=0.817; p=0.577), CI (p=0.817; p=0.214), and STR (p=0.817; p=0.469).

Discussion

The observed correlations of the assessed hemodynamic parameters with BNP/NT-proBNP levels correspond to the theoretical assumptions about patients presenting with heart failure. A positive relationship between TFC and BNP/NT-proBNP can be explained by intrathoracic fluid retention, which leads to pulmonary congestion causing a decrease in thoracic impedance. In clinical practice, measurements of thoracic bioimpedance are routinely applied to plan renal replacement therapy, which is used to quantify the degree of fluid overload and to guide therapeutic fluid depletion [31]. Theoretically, the same principles could be applied to decongestive heart failure, as they may help adjust diuretic treatment in addition to conventional monitoring parameters such as BNP/NT-proBNP, creatinine, or body weight.

The inverse relationship of BNP/NT-proBNP with SV and CI can be related to reduced myocardial contractility of ischemic or non-ischemic origin and fluid overload. There is substantial evidence for an increase in BNP/NT-proBNP with decreasing myocardial contractility, elevated left ventricular pressure levels, and myocardial wall stress [32]. An assessment of SV would also contribute to an improved understanding of the underlying pathophysiology of heart failure by differentiating normal from low-output states [33]. Measurements of systolic time intervals, including PEP, LVET, and their ratio (STR), are particularly useful for investigating the contractile state of the myocardium. Different clinical studies have shown that an increasing STR is related to a decrease in left ventricular systolic function, while a decreasing STR indicates diastolic dysfunction. The positive correlation with STR reflects a stronger influence by decreasing left ventricular systolic than diastolic function in the included study population [34,35]. Since the STR is defined as the ratio of PEP/LVET, a prolongation of the PEP or shortening of LVET would cause an increase in STR. A prolonged PEP is commonly related to reduced myocardial contractility due to ischemic or dilated cardiomyopathy and ventricular asynchrony [36,37]. This corresponds with the finding that all included studies reported a history of either known or suspected congestive heart failure. In addition, the PEP is often used as a surrogate parameter to measure myocardial sympathetic activity [38]. Although there is only limited data reported on the relationship with LVET, a shortened LVET is associated with left ventricular dysfunction and would contribute to an increase in STR [39,40]. Overall, the observed correlations confirm the theoretically presumed relationship between BNP/NT-proBNP and ICG parameters. The results of this meta-analysis are also compatible with earlier studies, which showed that ICG may help to distinguish systolic from diastolic heart failure [41] and differentiate cardiac from non-cardiac causes of dyspnea [42]. Measurements with ICG may also help to differentiate between hemodynamic states and, thereby, decide on an appropriate treatment strategy. Its application is user-independent, inexpensive, and carries a low procedural risk compared to echocardiography or invasive techniques. The ICG parameters are immediately accessible and allow for a comprehensive hemodynamic assessment for which physicians would have to apply different diagnostic methods in routine clinical practice.

However, the correlations were only moderate, and there was significant heterogeneity among the included studies, which may reduce the generalizability of the results. In addition, only four studies [24,27,29,30] assessed the ICG parameters at hospital discharge and none of them correlated these with BNP/NT-proBNP levels. Therefore, there is certainly a need for more research to investigate the utility of ICG for monitoring heart failure treatment.

## Conclusions

The results of this systematic review and meta-analysis show that ICG provides a set of hemodynamic parameters that may support clinicians in making the diagnosis and guiding the treatment of acute heart failure. By summarizing studies correlating BNP/NT-proBNP levels with ICG measurements, statistically significant positive relationships for TFC and an inverse correlation with SVI, CI, and STR were found. Theoretically, the investigation of TFC seems to be particularly interesting to monitor decongestive treatment in cardiac failure as it reflects the degree of intrathoracic fluid retention. In addition, the assessment of SVI, CI, and STR may help to observe changes in patients' functional status and anticipate clinical treatment response. Although several studies have confirmed the validity of ICG, more research is needed to investigate the application of bioimpedance in routine clinical practice and to raise awareness of its availability as a useful diagnostic tool in the context of acute heart failure.
